# Ground Reaction Forces and Throwing Performance in Elite and Novice Players in Two Types of Handball Shot

**DOI:** 10.2478/hukin-2014-0006

**Published:** 2014-04-09

**Authors:** Elissavet Rousanoglou, Konstantinos Noutsos, Ioannis Bayios, Konstantinos Boudolos

**Affiliations:** 1 Sport Biomechanics Lab-Department of Sport Medicine & Biology of Exercise, Faculty of Physical Education & Sports Science, National & Kapodistrian University of Athens, Greece.; 2 Department of Sport Games, Faculty of Physical Education & Sports Science, National & Kapodistrian University of Athens, Greece.

**Keywords:** braking force, postural control, jump shot, drive leg, injury prevention, handball

## Abstract

The purpose of this study was to examine the differences in the ground reaction force (GRF) patterns between elite and novice players during two types of handball shots, as well as the relationships between throwing performance and the GRF variables. Ball velocity and throwing accuracy were measured during jump shots and 3-step shots performed by 15 elite and 15 novice players. The GRF pattern was recorded for the vertical and the anterior-posterior GRF components (Kistler forceplate type-9281, 750Hz). One-way ANOVA was used for the group differences and the Pearson coefficient for the correlation between throwing performance and GRF variables (SPSS 21.0, p ≤ 0.05). The elite players performed better in both types of shot. Both groups developed consistent and similar GRF patterns, except for the novices’ inconsistent Fz pattern in the 3-step shot. The GRF variables differed significantly between groups in the 3-step shot (p ≤ 0.05). Significant correlations were found only for ball velocity and predominantly for the novice players during the 3-step shot (p ≤ 0.05). The results possibly highlight a shortage in the novice ability to effectively reduce their forward momentum so as to provide a stable base of support for the momentum transfer up the kinetic chain, a situation that may predispose athletes to injury.

## Introduction

Throwing performance in handball is typically evaluated with ball velocity and throwing accuracy (Bayios et al., 2001; García et al., 2013; Gorostiaga et al., 2005; van den Tillaar and Ettema, 2006; Wagner et al., 2011; Wagner et al., 2012). Based on the premise that the lower extremity drives the upper extremity’s motor pattern (Wagner et al., 2011; Zattara and Bouisset, 1998), throwing performance may be considered as the final outcome of an efficient kinetic chain. Previously, it has been reported that there is a significant relationship between ball velocity and the ground reaction forces (GRF) of the drive leg during softball pitching (Oliver and Plummer, 2011), as well as to the time to peak the vertical and braking GRFs during baseball (Elliot et al., 1988) and softball pitching (Guido and Werner, 2012). Similarly, MacWilliams et al. (1998) reported a significant relationship between linear wrist velocity and vertical, braking and resultant GRFs during baseball pitching. MacWilliams et al. (1998) did not measure ball velocity in all their subjects but they reported a high correlation of wrist and ball velocities for a single subject. Handball is a well-studied activity; however, there appears to be a lack of information about the GRFs developed during handball shots, as well as their relationship with throwing performance.

The GRFs reported for baseball (Elliot et al., 1988; MacWilliams et al., 1998) and softball pitchers (Guido and Werner, 2012; Oliver and Plummer, 2011) are considered to be large enough to predispose to injury (Oliver and Plummer, 2011). With the high risk of injury in handball (Junge et al., 2006), information about the GRFs developed during handball shots is warranted. The most frequent shots in handball are the jump shot (JS) and the 3-step shot (3SS). Their fundamental difference is that the upper extremity’s throwing movement is executed in the aerial phase for the JS whereas in ground contact for the 3SS (Wagner et al., 2011). As seen in throwing activities that are similar to 3SS, such as the javelin throwing (Whiting et al., 1991) and the baseball pitching (MacWilliams et al., 1998; Matsuo et al., 2001), the braking action of the drive leg is essential to provide a stable base of support for the transfer of momentum through the pelvis and trunk to the throwing arm. Thus, it may be assumed that the relationship between the GRFs and throwing performance is greater in the 3SS than in the JS.

The purpose of this study was to examine the differences of the GRF pattern developed by elite and novice players during the handball JS and 3SS, as well as the relationship between the GRF pattern variables and the throwing performance variables (ball velocity and throwing accuracy).

## Material and Methods

### Participants

The elite group (EG) included 15 males among the best scorers in the 1^st^ division of the Handball National League with a training experience of 12.3 ± 3.0 years (age: 24.9 ± 2.9 years, body height: 181.3 ± 6.3 cm, body mass: 83.1 ± 5.3 kg). The novice group (NG) included 15 male students of physical education and sport science who had completed a handball course (4 months, 3 hours per week) (age: 21.7 ± 0.9 years, body height: 181.7 ± 5.5 cm, body mass: 77.1 ± 6.4 kg). All participants were free of medical problems or pain for at least the past 6 months. All subjects signed an informed consent form that described the testing procedure in detail. The work reported was approved by the institutional review board and conformed to the principles outlined in the Declaration of Helsinki.

#### Procedures

A 15-min warm-up was allowed for each participant including general and shoulder-specific mobility exercises, as well as stretching exercises and familiarization with the protocol. Participants were instructed to complete five trials from the 7 m penalty line for both the JS and 3SS using a standard official ball (0.44 kg, 58.1 cm). Participants were allowed 1 min rest between trials. The trial with the greatest ball velocity was selected for further analysis. If the ball velocity was the same in two or more trials, the one with the best throwing accuracy was selected.

The ball velocity was measured by an innovative electronic device described in detail by Bayios et al. (2001). Briefly, the device consisted of a laser beam emitter and an electronic system laser beam infrared detectors, which were connected to a digital pulse counter. The ball interrupted the laser beam at a distance of 1.5 m after the penalty line. The ball velocity, which was calculated by multiplying the beam interruption time by the ball’s diameter, was expressed in meters per second (m/s).

The accuracy of the shot was measured by an innovative electronic device described in detail by Bayios et al. (1998). Briefly, the device consisted of a Π-shaped tabloid surface that was attached firmly to the inner side of a handball goal post. The tabloid surface included a net of light-emitting diodes (LEDs) (target hit pointers) that were interwoven with a net of metal strips (hit point detectors). The hit point detectors transferred the coordinates of the actual hit point to the central unit with 1 mm accuracy. Throwing accuracy was defined by the difference between the coordinates of the target and the actual hit point. The player initiated his trial when the target-pointer lit up (randomly via an electronic programmer). Trials in which the tabloid surface was not hit were rejected and additional trials were conducted until a total of five successful shots were achieved.

GRFs were recorded when the drive leg landed on the forceplate (60 × 40 cm, Kistler type – 9281, 750 Hz mounted flush with the floor at the 7 m penalty position, Bioware software Kistler). The GRF data were filtered (10 Hz low pass Butterworth filter, Bioware software Kistler). The GRF variables inserted for analysis were the contact time (t_contact_) expressed in milliseconds (ms), the peak vertical (Fz_max_) and anterior-posterior (Fy_max_) GRF components expressed as a multiple of body weight (BW), the time to peak Fz_max_ and Fy_max_ (tFz_max_ and tFy_max_, respectively) expressed in milliseconds and as a percentage of t_contact_ (% t_contact_) and the vertical and anterior-posterior impulses (Fz_impulse_ and Fy_impulse_, respectively) expressed in BW·s units.

#### Statistical Analysis

One-way ANOVA was used to test the group differences (EG versus NG) in the GRF pattern variables (t_contact_, Fz_max_, Fy_max_, Fz_impulse_, Fy_impulse_, tFz_max_, tFy_max_) and the throwing performance variables (ball velocity and throwing accuracy) separately for each type of shot. The Pearson Coefficient of Correlation was used to test the significance of the relationships between the GRF and the throwing performance variables in the EG and NG separately for each type of the shot. The level of significance was set at p ≤ 0.05 for all statistical tests (SPSS 21.0).

## Results

The mean values and standard deviations of the throwing performance and GRF variables of both types of shots for the EG and NG are presented in Table 1. The EG had greater ball velocity and better throwing accuracy in both types of shot (p ≤ 0.05) (Table 1).

**Figure 1.**
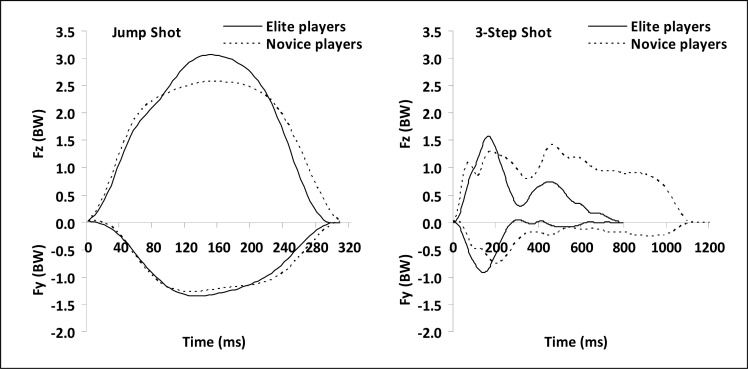
Representative GRF patterns for the vertical GRF (Fz) and the anterior-posterior (Fy) GRF components during the Jump Shot (Left) and the 3-Step Shot (Right) for the elite (solid line) and novice players (dotted line), respectively. Note: The negative values for the Fy component indicate the braking activity of the drive leg.

The GRF patterns developed by the EG and the NG in the JS and the 3SS appear in Figure 1. In the JS, the EG and the NG present rather similar GRF patterns. In specific, consistently in both groups and with no significant group differences (p > 0.05), the Fz and Fy force components exhibited a gradual increase to Fz_max_ and Fy_max_, until about 50% and 43% of t_contact_, respectively. After their maximum peak they gradually dissipated to the end of t_contact_ which was not significantly different between the EG and NG (p > 0.05). The Fz_max_ as well as the Fz_impulse_ were significantly greater in EG indicating their greater effort for the vertical propulsion of their body mass (p ≤ 0.05). The negative sign of the Fy force component throughout t_contact_ indicates the continuous application of a braking force in both the EG and the NG. The group similarity in the application of the braking force is further evidenced in the absence of significant Fy_max_ and Fy_impulse_ differences between the EG and the NG (p > 0.05).

In the 3SS the two groups did not present similar GRF patterns. Both the Fz and Fy force components were developed in significantly longer t_contact_ in NG than EG (p ≤ 0.05). This finding together with the non-significant group differences in Fz_max_ and Fy_max_ (p > 0.05) indicate that the significantly greater Fz_impulse_ and Fy_impulse_ of the NG (about 1.7 and 1.8 times greater than EG, respectively) could be due to their longer t_contact_ rather than the greater force application. The Fz force component developed a dissimilar pattern in the NG and the EG. In the NG, its maximum peak was reached significantly later than in the EG (p ≤ 0.05) and it was not always the first peak developed immediately after foot contact as occurred in the EG. The variation in the absolute and the relative tFz_max_ in the NG was reflected in the respective standard deviations which were about 8 and 4 times greater, respectively, than those of the EG. The Fy force component consistently rised to its maximum peak after foot contact without significant group differences (p > 0.05). After its peak, the Fy force component dissipated and fluctuated at around 0 BW and −0.2 BW in the EG and NG, respectively, for the rest of t_contact_. The Fy force component maintained a negative sign indicating a continuous braking impulse in the direction of the shot throughout t_contact_.

The coefficients of correlation of the GRF variables and the throwing performance variables are shown in Table 2. In regard to ball velocity, significant correlations were found in the NG and only in the 3SS (Table 2). They show an increase in ball velocity when there was an increase of Fz_max_ and Fy_max_ and a decrease of t_contact_ and tFy_max_. The only significant correlation in the EG was an increase in ball velocity when tFy_max_ was decreased. There were no significant correlations of throwing accuracy to GRF variables (p > 0.05).

## Discussion

The purpose of this study was to examine the differences in the GRF pattern between EG and NG during the handball JS and 3SS, as well as the relationship between GRF pattern variables and throwing performance variables (ball velocity and throwing accuracy). As expected, the ball velocity (Bayios et al., 2001; Gorostiaga et al., 2005; van den Tillaar and Ettema, 2006; Wagner et al., 2011) and throwing accuracy (García et al., 2013; van den Tillaar and Ettema, 2006) were greater in EG than in NG, in both the JS (25% and 57% higher, respectively) and the 3SS (29% and 39% higher, respectively).

The EG developed greater vertical force (+14%) in both types of the shot, but their differences were significant only in the JS. To our knowledge, there have been no previous GRF data of handball shots with which to compare the findings of the present study. Lindner et al. (2012) used the GRFs developed during JS trials to calculate and visualize lateral ankle ligaments’ force scenarios. However, they provided no GRF data. The peak magnitudes of the GRFs developed during the 3SS are similar to the vertical and anterior-posterior forces reported for throws while in contact with the ground as occurs in the 3SS for men’s baseball pitching (1.5 BW and 0.7 BW, respectively) (MacWilliams et al., 1998) youth softball pitching (1.4 ± 0.4 BW and 1.2 ± 0.5, respectively) (Guido and Werner, 2012) and women’s softball pitching (1.8 ± 0.4 BW and 0.4 ± 0.1 BW, respectively) (Oliver and Plummer, 2011). GRFs of the magnitudes of those developed in our study during the 3SS are considered large enough to predispose to injury (Oliver and Plummer, 2011). The lower extremity movement has a clear relationship with core musculature activity with current evidence suggesting that decreased core stability may predispose to injury (Willson et al., 2005). Thus, the adequate strengthening of core musculature is endorsed for injury prevention so as to ensure the body’s ability to maintain or resume postural control (Kibler et al., 2006; Oliver and Plummer, 2011; Willson et al., 2005).

In both the JS and the 3SS throughout the contact phase, the drive foot developed a braking force which is in agreement with previous studies of javelin throwing (Whiting et al., 1991) and baseball pitching (MacWilliams et al., 1998; Matsuo et al., 2001). The braking action of the drive leg is considered essential to provide a stable base of support to transfer effectively the momentum to the throwing arm (MacWilliams et al., 1998; Matsuo et al., 2001; Whiting et al., 1991). The absence of a significant group difference in the anterior-posterior force in the 3SS would justify the postulation of a group similarity regarding the braking activity of the drive leg. However, this group similarity was not displayed for the vertical force. The difference in vertical force was evident in the variation of the time to reach the peak in the NG in both absolute and relative times (8 and 4 times greater than the EG, respectively). Thus, during the last stride, the NG does not manage to control their body weight support so as to effectively reduce their forward run-up momentum and thus achieving a balanced anchoring on the drive leg (MacWilliams et al., 1998; Wagner et al., 2012). Such a technical weakness of the NG may be reflected in their longer contact time (+34%), greater vertical (+57%) and anterior-posterior (+55%) impulses, together with similar relative times to peak the vertical and anterior-posterior forces, indicating their need for more time to stabilize the body for the energy transfer up the kinetic chain.

The dominance of significant correlations of ball velocity to GRF variables in the 3SS, but not in the JS, may be associated with the absence of ground contact during the upper extremity movement in the JS (Wagner et al., 2011). The absence of ground contact during the throwing movement requires a different strategy to enable the momentum transfer through the trunk to the throwing upper extremity and ultimately to the ball velocity (Wagner et al., 2011). The significant correlations of this study are in agreement with previous results in baseball (Elliot et al., 1988; MacWilliams et al., 1998) and softball pitchers (Guido and Werner, 2012; Oliver and Plummer, 2011) who threw with greater ball velocity when the vertical and anterior-posterior forces were greater and peaked in longer times. In the 3SS, a trend for increased ball velocity was evidenced when the vertical force peaked in longer time, however, the correlation did not reach statistical significance. The relationship between ball velocity and the time to peak GRFs has been associated with the efficiency of postural control during the braking action of the drive leg (Elliot et al., 1988; MacWilliams et al., 1998; Wagner et al., 2012). During this braking action, the player acts to decrease his forward momentum and create a stable base of support for the sequential segmental energy transfer and ball release. Thus, the finding of a significant increase in ball velocity when the anterior-posterior force peaked in longer time may be associated with the ability to drive the body over a stabilized leg through the braking action. Such a significant correlation was found only in the NG and only in the 3SS and may have implications for postural control training during the braking phase of the 3SS. From a conditioning, as well a rehabilitation standpoint, awareness of the body position and postural control is essential in dynamic sequential segmental motion (Kibler et al., 2006). Such a dysfunction within the kinetic chain may predispose athletes to injury since it may affect how forces are generated, summated, or transferred from proximal segments to the throwing upper extremity (Kibler et al., 2006).

## Conclusions

The main findings of the study are the group differences in the 3SS as well as the significant correlations between the NG throwing performance in the 3SS and the respective GRF variables. The NG developed an inconsistent pattern of vertical force with their longer contact time appearing as the more important group difference. Overall, the NG appear inefficient to create a stable base of support for transferring the energy up the kinetic chain most possibly due to an inadequate reduction of their forward momentum, a situation that may predispose to injury. Under the limitation that testing did not reflect the numerous situations of an actual team handball competition, the results could serve as a basis for the development of strategies that combine optimal technical training while minimizing the risk of injury, particularly for the NG.

## Figures and Tables

**Table 1:** Mean (SD) of the throwing performance variables and the GRF variables for the elite and novice players in the Jump Shot and the 3-Step Shot. The p values indicate the significance of the differences between the elite and novice players

	Jump Shot					3-Step Shot				
	
	Elite players		Novice players		p	Elite players		Novice players		p
	Mean	SD	Mean	SD		Mean	SD	Mean	SD	
Throwing performance variables										
Ball velocity (m/s)	23.2	2.1	17.5	1.5	0.00^*^	27.6	2.5	19.6	1.7	0.00^*^
Throwing accuracy	15.8	9.5	27.6	20.7	0.05^*^	16.3	10.2	41.4	19.9	0.00^*^
GRF variables										
t_contact_ (ms)	307	32	314	40	0.56	758	108	1,016	289	0.01^*^
Fz_max_ (BW)	2.9	0.3	2.5	0.5	0.01^*^	1.5	0.5	1.4	0.2	0.68
Fy_max_ (BW)	1.3	0.2	1.3	0.3	0.63	0.9	0.3	0.8	0.2	0.27
Fz_impulse_ (BW·s)	0.54	0.05	0.47	0.09	0.02^*^	0.47	0.18	0.82	0.23	0.00^*^
Fy_impulse_ (BW·s)	0.20	0.04	0.21	0.03	0.58	0.12	0.05	0.22	0.09	0.00^*^
tFz_max_ (ms)	157	27	156	36	0.96	153	28	292	191	0.02^*^
tFy_max_ (ms)	134	29	140	30	0.92	143	25	177	43	0.02^*^
tFz_max_ (% t_contact_)	50.8	4.8	49.4	7.6	0.56	20.9	4.2	29.3	17.6	0.12
tFy_max_ (% t_contact_)	43.6	6.7	42.9	6.6	0.80	18.5	3.0	17.9	3.6	0.65

* Significant differences between the elite and novice players at p ≤ 0.05

**Table 2:** Pearson coefficients of correlation (r) between ball throwing velocity and the GRF variables in the elite and novice players during the Jump Shot and the 3-Step Shot. The p values (p) for the significance of the correlations are also noted

	Jump Shot				3-Step Shot			
	
	Elite players		Novice players		Elite players		Novice players	
	
	r	*p*	r	*p*	r	*p*	r	*p*
t_contact_ (ms)	−0.30	0.28	−0.43	0.11	−0.44	0.10	−0.66	0.01^*^
Fz_max_ (BW)	0.30	0.28	0.45	0.09	0.46	0.09	0.75	0.00^*^
Fy_max_ (BW)	0.35	0.20	0.31	0.27	0.37	0.17	0.61	0.03^*^
Fz_impulse_ (BW·s)	0.16	0.57	0.11	0.70	0.43	0.11	−0.67	0.01^*^
Fy_impulse_ (BW·s)	0.12	0.68	0.12	0.67	−0.05	0.87	−0.28	0.36
tFz_max_ (ms)	−0.25	0.37	−0.33	0.24	−0.19	0.23	−0.52	0.07
tFy_max_ (ms)	−0.26	0.36	−0.48	0.07	−0.52	0.05^*^	−0.74	0.00^*^
tFz_max_ (% t_contact_)	−0.16	0.57	−0.11	0.70	−0.30	0.29	−0.21	0.50
tFy_max_ (% t_contact_)	−0.20	0.48	−0.33	0.23	−0.47	0.08	0.07	0.83

* Significant differences between the elite and the novice players at p ≤ 0.05
